# Efficacy of Ayurveda treatment protocol in the management of prediabetes- A randomized controlled clinical trial

**DOI:** 10.1016/j.jaim.2026.101392

**Published:** 2026-07-24

**Authors:** Suman Khanal, Suketha Kumari, S.D. Laxmikant, Asha

**Affiliations:** a Ayurveda and Alternative Hospital, Ratnanagar-01, Chitwan, 44202, Nepal; b Department of Kayachikitsa, KAHER's Shri B M Kankanawadi Ayurveda Mahavidyalaya, Shahapur, Belagavi, Karnataka, India; c Department of Shalya, KAHER's Shri B M Kankanawadi Ayurveda Mahavidyalaya, Shahapur, Belagavi, Karnataka, India

**Keywords:** Prediabetes, Lifestyle intervention, Integrated Ayurveda Treatment Protocol, Standard prediabetic care

## Abstract

**Background:**

Prediabetes is a subject of intermediate hyperglycaemia and a risk factor for various metabolic and cardiovascular diseases, which are silent in features. Early Screening and preventive measures in terms of diet and lifestyle modification can help reverse the prediabetic stage.

**Objective:**

To evaluate the efficacy of Ayurveda-based treatment protocol in the management of prediabetes and to compare it with the standard prediabetic care.

**Materials and Methods:**

A total of 40 patients meeting the criteria of HbA1C 5.7-6.4% and Indian diabetic risk Score of ≥60, with the age group of 18-70 years of either sex, participated in the study. They were randomly divided into two groups in which trial group, intervened with Integrated Ayurveda Treatment Protocol (IATP) contains *Triphala lauha churna* 3 g twice a day along with Ayurveda dietary and lifestyle intervention and control group received placebo powder 3 g twice a day for 90 days along with standard prediabetic care (SPC). The primary endpoints were changes in glycated hemoglobin (HbA1C), fasting blood glucose (FBG), postprandial blood glucose (PPBG), and secondary endpoints were lipid profile, diabetes quality of life (DQOL), and anthropometric measures.

**Results:**

Study data indicate that IATP showed significant improvement in HbA1C compared to the SPC (p < 0.001). IATP also showed significant improvement in FBG (p < 0.001), PPBG (p = 0.0003), total cholesterol (p = 0.0004), high density lipoprotein (p = 0.003), low density lipoprotein (p = 0.01), serum triglycerides (p = 0.003), serum creatinine (p = 0.0011) and DQOL (p < 0.001). Comparable outcomes were noted in weight (p = 0.24), BMI (p = 0.05), and waist-hip ratio (p = 0.06).

**Conclusion:**

IATP intervention showed significant improvement in glycaemic outcomes, lipid profile compared to the SPC group. Comparable outcomes were observed in anthropometric measures.

## 1 Introduction

Prediabetes, characterized by intermediate hyperglycemia and diagnosed through an HbA1C level of 5.7-6.4%, or by impaired glucose tolerance, is not only a precursor to diabetes but also linked to metabolic disorders such as metabolic syndrome and cardiovascular diseases [1]. Globally, prediabetes affects 7.3% of adults, with a prevalence of 34.6% in the U.S. and 14% in India [2,3]. Projections suggest that over 470 million people will have prediabetes by 2030 [4]. Since prevention is the mainstay of diabetes care, screening for prediabetic people is simple, and risk groups can be identified and monitored.

Ayurvedic texts describe the poorvaroopa (prodromal symptoms) of prameha, some of which may align with early clinical features observed in individuals with prediabetes, such as numbness in the feet and burning sensations. These parallel suggest a potential correlation between prameha purva roopa and the early stages of metabolic dysfunction [5]. However, a direct analogy requires cautious interpretation due to conceptual differences. A study suggests that Ayurveda medications, Ayurveda diet, panchakarma has shown beneficial effects [6]. Yoga practices showed beneficial effects in management of lifestyle disorders like prediabetes [7]. The present study focusses on Triphala lauha churna, a classical Ayurvedic formulation mentioned in Rasayoga sara for the management of Prameha. This formulation contains triphala, guduchi, haridra, daruharidra, and lauha bhasma, which are recognized for their antihyperglycemic, lipid-lowering, and antioxidant effects [8,9].

Hence, the present study is planned to evaluate the safety and efficacy of integrating Ayurveda treatment protocol (IATP), including Triphala Lauha Churna, a classical Ayurvedic Herbo-mineral formulation, alongside customized yoga and dietary interventions in the management of prediabetes, compared to standard prediabetic care (SPC). Exploring such complementary approaches may provide a more holistic and effective method for managing prediabetes.

## 2 Materials and methods

### 2.1 Research design

There was a randomized placebo-controlled study conducted at KLE Ayurveda Hospital, Belgaum. Random numbers for participant allocation were generated using the online tool Random.org (https://www.random.org/) created by Mads Haahr, Professor of computer science at Trinity college, Dublin, Ireland. The randomisation was performed on 18th February 2025 using the sequence generator function. No version number was applicable and accuracy data of the software is not available. A block design with 20 blocks of size 2 ensured an equal 1:1 allocation ratio between the control and trial groups. Allocation concealment was maintained using sealed opaque envelopes, which remained blinded to the investigators. An impartial assistant, not involved in patient allocation, unsealed the envelopes one by one after each patient consented to the study and complete their baseline assessment. Different members of the research team were responsible for managing the administration, distribution, and randomisation of study material.

Medicine adherence was assessed by counting the number of unused tablets returned during follow-up visits and by reviewing patient-maintained adherence diaries that recorded daily medication intake. Follow-up evaluations were conducted at 30-day intervals. Diet adherence was supported by providing patients with structured pdf diet plans. Daily reminders were sent, and patients were encouraged to share their food and exercise updates in a dedicated WhatsApp group. Additionally, a motivational social media group was created where daily motivational images or videos were shared to enhance patients’ engagement and compliance. Progress was monitored through periodic assessments of weight, glycaemic parameters, and overall health status, ensuring tailored feedback and adjustments when necessary.

The sample size calculation was conducted using previously published data [10] using mean fasting blood sugar (FBS), where group A had a mean FBS of 97.5 mg/dl (standard deviation (SD) = 13.5 mg/dl) and group B had a mean FBS of 107.8 mg/dl (SD = 8.7 mg/dl). Using these values, the sample size was calculated using formula N= (Zα/2 +Zβ)^2^ ×2/d/σ at 95% confidence interval and 80% power resulted in a required sample size of approximately 20 patients per group.

### 2.2 Participants

Patients were screened for the study from outpatient and inpatient department of KLE Ayurveda hospital, Belagavi, Karnataka. Individuals with an Indian Diabetes Risk score (IDRS) of ≥60, glycated haemoglobin (HbA1C) levels between 5.7% and 6.4%, fasting plasma glucose levels between 100 and 125 mg/dl, and who were fit for yoga were enrolled in the study.

Patients with history of cardiovascular accidents within the past six months, endocrine disorders (including microvascular complications of T2DM), thyroid dysfunctions, serious health conditions such as carcinomas or cardiovascular diseases, major psychiatric disorders, pregnant and lactating women, and those with uncontrolled hypertension (**≥** 140/90 mmHg) were excluded from the study.

**Fig. 1:**
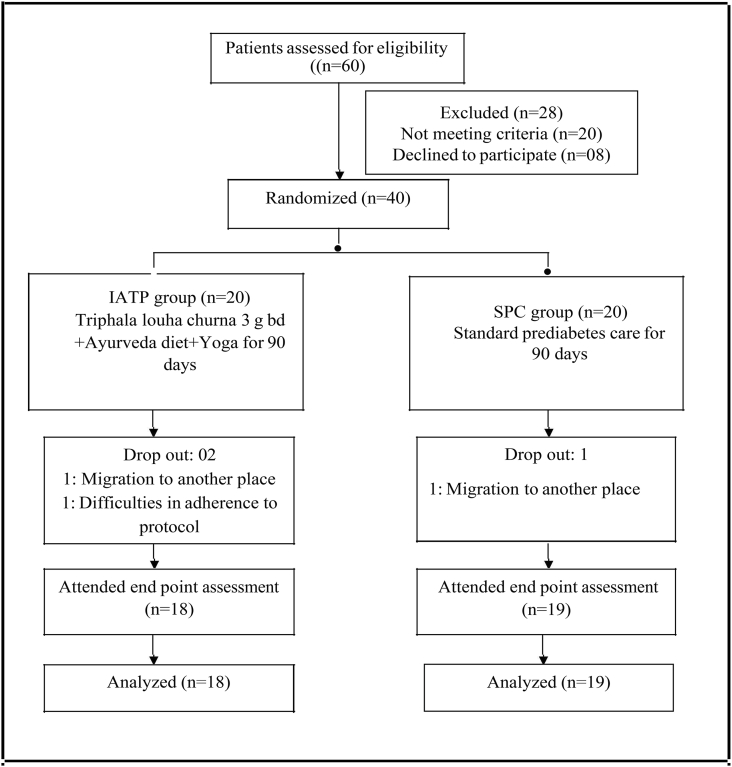
Subject flow diagram through the study.

The study adhered to ethical guidelines, with written informed consent from all participants. The study protocol was approved by the institutional ethics committee (protocol ID: BMK/19/PG/KC/06) and registered with the clinical trial registry India (CTRI/2020/11/028809). Data collection was conducted between July 2021 and March 2022. Patients were instructed to adhere to the treatment protocol and promptly report any adverse events to the research team. The study was conducted in accordance with the CONSORT guideline [11] [fig.1].

### 2.3 Intervention

According to the randomisation, each patient was randomly assigned to one of two interventional groups. SPC group received placebo of fried wheat flour 3 g twice daily with lukewarm water, along with standard prediabetic care. As the trial was open label, the placebo was used to maintain uniformity in administration and to control for behavioural bias. Fried wheat powder is inert and safe. This helped to evaluate whether standard care alone is sufficient, or additional intervention offers superior benefit. Along with this, structured approach combining medical nutrition therapy and physical activity was included as a part of standard prediabetic care. Patients were guided to reduce their daily intake by 500-1000 kcal based on their energy requirements. Nutrient rich food was included in appropriate portions, ensuring a balanced intake of carbohydrates and proteins from fruits, legumes, whole grains, and dairy products. Dietary fat consumption was minimised, with particular emphasis on reducing saturated and *trans*-fat. Sugar sweetened beverages and added sugars were restricted, while high fiber foods were encouraged-targeting 14 g of fibre per 1000 kcal of energy intake. The dietary plan also included 5 to 7 servings of fresh fruits and vegetables per day. Patients advised to engage in moderate intensity physical activity for at least 150 min per week. These activities were distributed across three or more days, ensuring no more than two consecutive days without exercise to maintain consistent metabolic benefits. The programme also emphasized the inclusion of resistance training at least twice a week to enhance muscle strength and improve glucose uptake.

The IATP group (IATP) received the integrated protocol which include polyherbal medicine i. e Triphala Lauha Churna (TLC) 3 g twice daily with lukewarm water along with integration of ayurveda diet protocol and therapeutic yoga. Loha Bhasma was procured from a GMP-certified Ayurveda pharmacy Pune. Other raw materials of TLC were sourced from the GMP-certified KLE Ayurveda Pharmacy, Belagavi. All materials were authenticated at the central research facility (CRF) of KLE BMK Ayurveda Mahavidyalaya, an AYUSH-approved drug testing laboratory for Ayurveda, Siddha, and Unani (ASU). Each raw material and the completed product underwent a qualitative examination following Ayurvedic Pharmacopoeia of India (API) requirements. Raw pharmaceuticals were analysed for ash, water, and alcohol-soluble extractive values, as well as drying loss. The churna of each drug was prepared according to the standard operating procedures outlined in the Ayurvedic formulary of India (AFI). The qualitative analysis of the finished product included assessments of ash values, drying loss, microbial studies, and tests for organic and inorganic materials.

The dietary plan was grounded in Ayurvedic principles, focussing on balancing the doshas and enhancing digestive health. Meal timings were synchronised with body's circadian rhythm-promoting the consumption of the largest meal between 10 a.m. and 2 p.m., when agni (digestive capacity) is considered strongest, a light, easily digestible dinner before sunset to avoid gastrointestinal burden during the kapha period. The diet emphasized satvik, wholesome foods and ensured inclusion of all six tastes in major meals to promote physiological balance. Breakfast featured easily digestible protein sources such as moong beans, lentils or sprouted legumes, prepared with mild digestive spices to stimulate agni. Lunch and dinner included seasonal millets such as jowar, bajra or barley paired with seasonal vegetables and digestive spices like cumin, coriander, and turmeric. Plates were portioned to promote satiety and nutritional adequacy-half comprised of steamed or sauteed vegetables, one quarter protein rich food (legumes, seeds, paneer), and the remaining quarter composed of whole grains, with moderate use of ghee to support digestion and flavour. Low glycaemic index fruits and non-starchy vegetables formed the foundation of the diet to minimize glycaemic load and stabilize blood glucose levels. Starchy vegetables and grains were in controlled quantities. Soluble fibre was primarily sourced from whole grains, pulses, seeds, and a variety of vegetables, supporting gut health and glycaemic control. Morning beverages included warm herbal infusions with ingredients like ginger or cinnamon, and green smoothies enhanced with fresh herbs. Healthy fats were obtained from sources such as flaxseeds, fenugreek, and ghee, which were incorporated into chutney's, spicy dips or as tempering agents. Ghee was used in moderation for its digestive and therapeutic potential **(Supplementary file.1)**.

Yogic intervention included body loosening exercise, asanas, suryanamaskara, pranayama and meditation conducted under the guidance of a trained yoga instructor. Patients underwent 15 days residential training programme where they were taught proper techniques and encouraged to practice consistently. The session focussed on improving flexibility, strength and mindfulness **(Supplementary file.2).**

Lifestyle interventions in both groups were structured rather than personalised. In the IATP group, dietary recommendations were based on Ayurvedic principles with standardised calorie specific charts, while in the SPC group, a standard ADA based diet guideline was adapted to suit local dietary practices.

### 2.4 Primary outcome measures

The primary outcome variables were Glycated haemoglobin (HbA1c) (%), Fasting blood glucose (FBG in mg/dl), Post prandial blood glucose (PPBG in mg/dl).

### 2.5 Secondary outcome measures

Lipid profile(mg/dl), weight(kg), BMI (kg/m^2^), waist circumference, waist hip ratio, and diabetes quality of life (DQOL) were the secondary outcomes.

HbA1C, lipid profile, serum creatinine was assessed on 0^th^ and 90th day of intervention. Weight, BMI, waist circumference, waist hip ratio, FBG, PPBG and DQOL were assessed on 0^th^,30th,60th and 90th day of intervention. The glucose oxidase and peroxidase (GOD-POD) method was used to estimate FBG and PPBG. The turbidimetric inhibition immunoassay (TINIA) was used to measure HbA1C. The lipid panel components, including total cholesterol and high-density lipoprotein (HDL) levels were measured using cholesterol oxidase p-amino phenazone (CHOD-PAP) method. Serum triglyceride levels were assessed using catachem triglyceride method. Low density lipoprotein (LDL) and very low-density lipoprotein levels (VLDL) cholesterol levels were calculated using fried Wald equation.

### 2.6 Statistical analysis

SPSS Version 25.0 (IBM Corporation, Chicago, Illinois, United States) was used for statistical analysis. The homogeneity between groups was evaluated using the χ2 test. Group comparisons at specific time points were conducted using the independent *t*-test, while comparisons within the group at specific time points were conducted using the dependent *t*-test. The results are presented as the mean difference between the groups, along with the corresponding 95% confidence interval (CI). All tests were considered statistically significant at p < 0.05. The Cohen's d method was used to estimate the effect size, with the following criteria for interpretation: 0–0.2 as the smallest effect size, 0.2–0.5 as modest, 0.5–0.8 as medium, and over 0.8 as large.

## 3 Results

A total of 40 patients were enrolled in the study. In IATP group, two patients discontinued treatment, one due to relocation and the other because of difficulty adhering to the protocol. Similarly, one patient in the SPC group dropped out after relocating to a different location [Fig. 1].

### 3.1 Patient profile

The study shows that IATP and SPC groups were similar in terms of age (p = 0.54), gender (p = 0.445), religion (p = 0.30), occupation (p = 0.847), socio-economic status (p = 0.263), education status (p = 0.639), prakriti (p = 0.79) and family history of T2DM (p = 0.228)**.** Physical activity wise distribution of subject revealed that moderate (n = 6 in IATP and n = 10 in SPC) to sedentary type (n = 10 in IATP and n = 8 in SPC) of lifestyle was observed (Table 1). Between the group, this change was non-significant (p = 1.89). A non-significant difference was observed between the group in diet (p = 2.93) and yoga/exercise adherence (p = 1.01). However, group wise observation of adherence in percentage showed that higher percentage of adherence was observed in IATP (diet = 94%, exercise = 95%) than SPC (diet = 75%, exercise = 86%).

**Table 1:** Baseline characteristics (n = 40).

Clinical Profile	-	IATP	SPC	P value
Age (years)		47.22 ± 13.25	44.63 ± 12.74	0.54
Gender	Male: Female	12: 6	10:9	0.445
Occupation	Unemployed Skilled and Market workers Professionals Technicians Machine operators Senior officers Craft related trade worker Elementary Occupation Clerks	6 4 3 2 1 1 1 0 0	6 0 3 2 0 1 1 4 2	0.847
Socio-economic Status	Middle: Lower: Upper	15:1:2	13:3:3	0.263
Education Status	High School Graduate Primary school Diploma Postgraduate Illiterate	5 5 4 2 2 0	5 4 4 4 1 1	0.639
Diet	Vegetarian Mixed	10 8	10 9	0.7616
Prakriti	Kapha vata Kapha pitta Vatakapha Pittakapha Vatapitta Pittavata	6 4 3 3 1 1	7 5 3 3 1 1	0.79
Family history of T2DM	Yes No	6 12	9 10	0.228
Physical activity/lifestyle factor	Sedentary Moderate Active	10 6 4	8 10 2	1.89
Diet adherence (%)	-	94%	75%	2.93
Yoga/Exercise adherence (%)	-	95%	86%	1.01
Study completed	-	18	19	-

*Statistical significance using Chi-square test expressed as p < 0.05.

### 3.2 Primary outcomes

#### 3.2.1 Glycated haemoglobin [HbA1c]

Effect of intervention on HbA1C revealed that, IATP showed significant difference compared to SPC group [p < 0.001, mean difference(md): -0.83, 95% confidence interval (CI): -1.04 to -0.61] and effect size was high. In IATP, mean change from before to after intervention was 6.08 ± 0.28 to 5.53 ± 0.22, where as in SPC group there was increase in mean changes from 5.88 ± 0.25 to 6.36 ± 0.39. These findings indicate that HbA1C levels improved in the IATP group, while an increase was observed in the SPC group during the study period (Table 2).

**Table 2:** Effect on outcome variables- Glycemic parameters, lipid profile, serum creatinine, anthropometric measures and DQOL.

S·N	Parameters	Group	Baseline	30th day	60th Day	90th Day	Mean difference	95% confidence interval	P-value between the group^a^	Effect size
1.	Weight (kg)	IATP	80.74 ± 9.20	79.88 ± 9.01	79.23 ± 9.04	78.45 ± 9.02	3.45	2.53 to 9.43	0.24	0.73
		SPC	73.85 ± 8.58	73.94 ± 9.00	74.29 ± 8.85	75.00 ± 8.90				−0.27
3.	BMI (kg/m^2^)	IATP	27.33 ± 1.79	27.16 ± 1.72	26.87 ± 1.63	26.04 ± 1.50	−1.13	−2.31 to 0.04	0.05	0.6
		SPC	27.04 ± 1.93	27.06 ± 2.05	27.17 ± 2.02	27.53 ± 1.99				−0.2
5.	Waist circumference (cm)	IATP	86.08 ± 6.31	85.87 ± 6.26	85.69 ± 6.26	85.35 ± 6.39	−0.72	−4.73 to 3.28	0.71	0.2
		SPC	85.47 ± 5.54	85.79 ± 5.50	85.96 ± 5.69	86.08 ± 5.62				−0.4
6.	Waist hip ratio	IATP	0.83 ± 0.06	0.83 ± 0.06	0.82 ± 0.07	0.82 ± 0.06	−0.72	−4.73 to 3.28	0.06	0.2
		SPC	0.86 ± 0.08	0.86 ± 0.08	0.87 ± 0.08	0.86 ± 0.08				−0.3
8.	FBG (mg/dl)	IATP	92.78 ± 10.88	90.78 ± 9.71	86.94 ± 6.83	83.50 ± 5.69	−17.55	−24.89 to 10.20	<0.001***	0.36
		SPC	88.84 ± 11.18	92.16 ± 8.02	95.21 ± 8.68	101.05 ± 14.30				−0.38
10.	PPBG (mg/dl)	IATP	105.00 ± 17.84	102.17 ± 11.19	102.61 ± 10.05	95.56 ± 6.50	2.63	−9.43 to 14.69	0.0003**	0.2
		SPC	102.37 ± 18.29	108.37 ± 18.13	113.32 ± 13.45	118.84 ± 23.68				−0.8
12.	HbA1C (%)	IATP	6.08 ± 0.28	-	-	5.53 ± 0.22	−0.83	−1.04 to -0.61	<0.001***	2.18
		SPC	5.88 ± 0.25	-	-	6.36 ± 0.39				−1.47
14.	TC (mg/dl)	IATP	194.00 ± 32.12	-	-	173.06 ± 17.28	−26.52	−44.38 to -8.66	0.004*	0.81
		SPC	200.42 ± 39.69	-	-	199.58 ± 33.30				−0.02
16.	HDL (mg/dl)	IATP	45.06 ± 5.37	-	-	49.33 ± 4.83	5.43	−44.38 to -8.66	0.003	0.8
		SPC	45.53 ± 4.17	-	-	43.89 ± 5.61				−0.3
18.	LDL (mg/dl)	IATP	127.78 ± 27.63	-	-	116.39 ± 18.06	−22.24	−39 to -5.48	0.0178*	0.49
		SPC	134 ± 37.44	-	-	138.63 ± 30.28				−0.14
20.	TGL (mg/dl)	IATP	138.61 ± 55.23	-	-	120.61 ± 29.40	−36.59	−60.62 to -12.57	0.003*	0.41
		SPC	150.00 ± 51.10	-	-	157.21 ± 41.25				−0.16
22.	Sr. Creatinine (mgs%)	IATP	0.76 ± 0.10	-	-	0.72 ± 0.09	−0.09	−0.177 to −0.003	0.0011**	0.42
		SPC	0.76 ± 0.14	-	-	0.81 ± 0.17				0.32
24.	DQOL	IATP	100.17 ± 16.05	90.61 ± 12.87	83.44 ± 12.10	76.50 ± 10.88	−44.4	−52.34 to -36.55	<0.001***	0.8
		SPC	104.16 ± 8.32	107.11 ± 10.36	115.05 ± 9.92	120.95 ± 12.65				0.6

Expressed in terms of the mean value, standard deviations (SD), mean difference and 95% confidence interval. *p < 0.05, **p < 0.01, ***p < 0.001.

a Statistical significance at before and after the intervention between the group with unpaired ‘t’ test.

#### 3.2.2 Fasting blood glucose (FBG in mg/dl)

Study outcome revealed that, IATP showed significant difference compared to SPC [p < 0.001, md: -17.55, 95% CI: -24.89 to -10.20]. IATP group had significant difference from baseline to 60th (p = 0.02) and 90th (p = 0.0005) day of intervention in group analysis. Effect size was large (Table 2).

#### 3.2.3 Post prandial blood glucose (PPBG in mg/dl)

Intervention impact on PPBG revealed that IATP is better than SPC in between the group comparison [p = 0.0003, md: 2.63, 95% CI: -9.43 to 14.69]. Effect size was large (Table 2).

### 3.3 Secondary outcome

#### 3.3.1 Lipid panel parameters (in mg/dl)

Interventional impact on total cholesterol showed that, there is significant difference between the group favouring towards IATP [p = 0.004, md: -26.52, 95% CI: -44.38 to -8.66]. IATP also showed better results than SPC in HDL [p = 0.003, md: 5.43, 95% CI: -44.38 to -8.66], LDL [p = 0.01, md: -22.24, 95% CI: -39 to -5.48] and triglyceride parameters [p = 0.003, md: -36.59, 95% CI: -60.62 to -12.57] (Table 2).

#### 3.3.2 Serum creatinine (in mgs%)

Study found that, serum creatinine levels were comparable between the group. However, within the group both IATP (p = 0.04) and SPC (p = 0.008) showed significant difference (Table 2).

### 3.4 Anthropometric measures

#### 3.4.1 Weight in (Kg's) and BMI

Impact of intervention on weight parameter revealed that there is comparable outcome between the group [p = 0.24, md: 3.45, 95% CI: -2.53 to 9.43]. Within the group, IATP showed significance on 30th (p = 0.001), 60th (p = 0.0002) and 90th day (p < 0.001) of intervention. In SPC group, we observed there is mean weight of 2 kg increase before to after intervention (Table 2).

In BMI parameter also, there is comparable outcome between the group [p = 0.05, md: -1.13, 95% CI: -2.31 to 0.04]. Within the group, IATP showed significant difference from 30th(p = 0.001), 60th (p = 0.0002) and 90th day(p < 0.001)) of intervention (Table 2).

#### 3.4.2 Waist circumference

Impact of intervention on waist circumference revealed that, there is comparable outcome between the group [p = 0.71, md: -0.72, 95% CI: -4.73 to 3.28] (Table 2).

#### 3.4.3 Waist-hip ratio

Result showed that, in waist hip ratio before to after intervention there is similar finding between IATP and SPC group [p = 0.06, md: -0.72, 95% CI: -4.73 to 3.28](Table 2).

### 3.5 Diabetes quality of life (DQOL)

IATP showed statistical significance compared to SPC in DQOL in all time intervals [p < 0.001, md: -44.4, 95% CI: -52.34 to -36.55]. Within the group, results at different time points revealed that significant changes in IATP observed on 30th(p < 0.001),60th (p < 0.001), and 90th (p < 0.001) day of intervention. In SPC group, there is negative increase in mean value was observed with statistical significance showed on 60th (p < 0.001) and 90th (p < 0.001) day of intervention (Table 2).

## 4 Discussion

The study demonstrated that the IATP group which consists of Ayurvedic polyherbal formulation, diet, and yoga intervention was more effective than the SPC group which consists of standard prediabetic care. The IATP group showed greater efficacy in primary assessment parameters, including HbA1C, FBG and PPBG. Additionally, the IATP group outperformed the SPC group in secondary assessment parameters such as lipid profile, serum creatinine and DQOL. Comparable outcome observed in anthropometric measures.

The primary outcome demonstrated a significant impact on the IATP group, effectively reversing prediabetes as indicated by HbA1C levels below 5.7%. In contrast, the SPC group experienced a significant rise in HbA1C, though it did not reach diabetic levels in terms of FBG and PPBG.

The protocol for the IATP group included the Herbo-mineral formulation Triphala Lauha churna, which contains *Terminalia chebula* Retz (fruit), Terminalia Billerica Roxb (fruit), Emblica officinalis Gaertn (fruit) and curcuma longa Linn(rhizome) which had Antioxidant [12,13], Immune modulator [14] potential. *Berberis aristata* DC (stem) had anti-diabetic and lipid-lowering action [15] might acted in prediabetic and lipid parameters. *Tinospora cordifolia* Willd (leaves) has anti-inflammatory [16] properties contributed to glycaemic control and insulin resistance. Research has indicated that triphala act as a potential medicinal agent for reducing body fat and aiding in weight loss [17,18].

The dietary intervention implemented in this study was rooted in Ayurvedic principles, and the inclusion of nutrient dense wholesome foods such as lentils, beans, millet-based grains, and vegetables. Herbal infusions complemented this dietary approach, promoting better digestion and metabolic health. This integrative dietary therapy aligns with traditional practices while addressing nutritional needs, creating a foundation for assessing its impact in clinical outcomes.

Dietary interventions based on Ayurvedic principles, including the use of antidiabetic spices and small, frequent meals, have also been effective in controlling blood sugar levels [19]. Some randomized controlled trials show that low-carbohydrate diets prevent body weight more effectively than low-fat diets [20,21]. A reduced food consumption window of 10 h/day (14 h of fasting) helps in weight loss in patients having diseases such as type 2 diabetes, metabolic syndrome & pre-diabetes. It decreases waist circumference, visceral fat, blood pressure, atherogenic lipoproteins, and HbA1C [22]. A daily food consumption window reduced to 4 h, or 6 h/day (20 h or 18 h of fasting) resulted in a 3.2% loss of body weight while improving fasting insulin levels, insulin resistance, and oxidative stress [23]. Millets with a high soluble dietary fibre content increase glucose tolerance by reducing the amount of glucose absorbed and the rate at which the stomach empties after a meal [24]. Traditional snacks i. e lotus seeds used in the diet contain alkaloids and flavonoids which helped in anti-obesity and hypolipidemic properties [25]. We strictly restricted refined carbohydrates, sugar, packed and processed foods, and most of the fatty fried foods. Yoga results in parasympathetic activation, which reduces stress and improves insulin sensitivity, glucose tolerance, lipid metabolism, and overall metabolic and psychological improvements [7,26]. Yoga has been shown to positively influence type 2 diabetes management by improving insulin production and stimulating pancreatic function through practices like Surya Namaskar, *Tadasana, Kapalbhati,* and *Agnisara* [27]. This therapeutic plan helped in achieving good glycaemic control in terms of glycosylated haemoglobin, weight, BMI, and overall quality of life.

### 4.1 Strength of study

The design being randomized and controlled with the incorporation of a holistic approach based on Ayurveda and yoga lifestyle is the major strength of the study. The usage of parameters like HbA1C, Lipid profile, creatinine, and DQOL are notable components of the study.

### 4.2 Limitations of study

A larger sample size with a longer duration of the study would have brought more strength to the study. The study can be replicated with standard diagnostic methods like oral glucose tolerance test, and insulin resistance parameters [serum insulin and homeostatic model for assessment of insulin resistance (HOMA IR)] in multicentric study designs.

## 5 Conclusion

This study demonstrated that the IATP had a notable impact on prediabetes management. Significant improvements were observed in glycemic markers such as HbA1C, fasting blood glucose, and post prandial blood glucose, alongside enhancement in diabetes quality of life. SPC resulted in increased HbA1C, while IATP significantly reduced, highlighting the effectiveness of integrative care in prediabetes. Compared to standard prediabetic care, the IATP intervention also yielded favourable effects on lipid profile. The integrative approach, incorporating the polyherbal Ayurvedic formulation *Triphala louha churna*, therapeutic yoga and a tailored diet effectively controlled prediabetes by improving glycaemic measures, addressing lipid imbalances, and reducing weight, BMI and waist circumference, while enhancing overall quality of life. Notably, no adverse events were reported during the study.

## Authors contribution

SKh: Methodology, Investigation, Data collection, Writing–original draft preparation, Visualization, Statistical analysis. SK: Conceptualization, Methodology, Formal analysis, Writing -Reviewing and Editing, Supervision, Validation, Data curation. LS: Data curation, Supervision, Validation, Formal analysis, Data curation. AS: Visualization, Writing - Original draft preparation, Data Collection, Writing - Reviewing and Editing.

## Declaration of generative AI in scientific writing

Nothing to disclose.

## Funding sources

This research did not receive any specific grant from funding agencies in the public, commercial, or not-for-profit sectors.

## Conflict of Interest

None.

## Data Availability

Data related to the study is available in the manuscript and supplementary file. The raw data is not available to the public due to ethical issues. Data access will be made available upon request to the corresponding author.
