# Supplementary material for: Efficacy of Ayurveda treatment protocol in the management of prediabetes- A randomized controlled clinical trial
Source: J Ayurveda Integr Med. 2026 Jul 24;17(4):101392. doi: 10.1016/j.jaim.2026.101392 (PMC13420484; doi:10.1016/j.jaim.2026.101392)
Supplement: Multimedia component 1 [file mmc1.docx]

**Supplementary file.1: Ayurveda diet sample menu plan in prediabetes**

| **Timing**  **Food Diary** | **Menu** | **Serving** |
| --- | --- | --- |
| **Morning Drink**  **B/w 6.30 am to 7 am**  **(Any One)** | 1. Herbal Infused Drink (Lemon Added) | 150 ml (1 glass) |
|  | 1. Ginger Lemon water | 100 ml |
|  | 1. Cinnamom water (lemon added) | 100ml |
|  | 1. Chia Water (Lemon added) | 100 ml |
| **8 am to 9 am**  **Breakfast options**  **(Any one)** | 1. Broken wheat vegetable upma (Daliya Upma) | 1 bowl/1 plate |
|  | 1. Besan Dosa (Tomato Omlette)   + Herbal Chatni  +Sambar | 1 in no |
|  |  | 2 tbsp |
|  |  | 1 wati |
|  | 1. Barley/Whole wheat chapati   + Herbal/coconut Chatni  +Sambar | 2 in no |
|  |  | 2 tbsp |
|  |  | 1 wati |
|  | 1. Millet Dosa/Idli   + Herbal/ coconut Chatni  +Sambar | 1 in no/2 in number |
|  |  | 2 tbsp |
|  |  | 1 wati |
|  | 1. Millet Vegetable Upma | 1 bowl/1 plate |
|  | 1. Moong dosa   + Herbal / coconut Chatni  +Sambar | 1 in no |
|  |  | 2 tbsp |
|  |  | 1 wati |
|  | Breakfast with Herbal /Cardomom/Green Tea without sugar | 1 cup |
| **Mid-Morning Snack**  **11.30 am to 12 pm** | 1. Medicated Buttermilk (Spiced with Ginger, Pepper, Rock salt and Jeera) | 1 glass (150 ml) |
| **Lunch**  **1pm to 2 Pm** | Mix Veg Salad (Raw-spiced with pepper powder and Rock Salt) | 1 bowl |
|  | +Jowar/Bajra Roti | 1 in number |
|  | +Vegetable Sabji | 1 wati |
|  | +Boiled Pulses Sabzi/Junka /moong Dal | 3/4^th^ katori |
|  | +Peanut/Methi/Flax Chatni | 1 tbsp |
|  | + Dahi (Added with Rock salt) | 1 cup |
| **Mid Evening Snack**  **4.30 pm to 5 pm**  (Option-Any one) | 1. Apple/Pear Fruit | 1 medium Size |
|  | +Almond | 6 |
|  | +Walnut | 3 |
|  | + Green Tea/Lemon tea without Sugar | 1 cup |
|  | 1. Boiled Chana chat | 1 cup |
|  | +Almond | 6 |
|  | +Walnut | 3 |
|  | + Green Tea/Lemon tea without Sugar | 1 cup |
|  | 1. Boiled Peanut Chat or unsalted peanuts | 1 plate/1 handful |
|  | + Green Tea/Lemon tea without Sugar | 1 cup |
| Dinner  (7 pm to 8 Pm)  (Option-Any one) | 1. Salad (spiced with lemon, pepper, rock salt) | 1 bowl |
|  | + Jowar /Bajra Roti  +veg Sabzi | 1 in no  ½ wati |
|  | +Millet Rice | 1 Katori |
|  | + moong dal | 1 wati |
|  | + Herbal Raita | ½ wati |
|  | 1. Salad (spiced with lemon, pepper, rock salt) | 1 bowl |
|  | + Moong Soup (spiced with Pepper and Rock Salt) | 1 bowl (150 ml) |
|  | + Jowar roti/Bajra roti  +veg Sabzi | 1 in no |
|  | +vegetable pulao | ½ bowl |
|  | + Herbal Raita | ½ wati |
|  | 1. Jowar roti/Bajra roti | 1 in no |
|  | + Vegetable Sabzi | 1 katori |
|  | + Moong millet Khichdi added with vegetables | 1 bowl |
|  | + Salad (spiced with lemon, pepper, rock salt) | 1 bowl |
