# Supplementary material for: Efficacy of Ayurveda treatment protocol in the management of prediabetes- A randomized controlled clinical trial
Source: J Ayurveda Integr Med. 2026 Jul 24;17(4):101392. doi: 10.1016/j.jaim.2026.101392 (PMC13420484; doi:10.1016/j.jaim.2026.101392)
Supplement: Multimedia component 2 [file mmc2.docx]

**Supplementary file.02.Yoga protocol**

| 1. **Surya Namaskara (5 minutes-12 steps)** |
| --- |
| 1. **Arda Kati chakrasana**   Stand straight, place your hands to your waist supporting the back. Draw your bent elbows backward keeping them parallel to each other. Inhale, stretching your neck drops the head backward. Exhale bending the upper body backward from the lumbar region. Maintain for 3-10 seconds and then release |
| 1. **Parivratta Trikonasana**   Inhale deeply and distance legs 3-4 feet apart. Raise your arms at shoulder level.  Exhale and turn the trunk to the right side, try to keep palm in the foot. Maintain for 3-4 seconds and return to normal. Repeat the same with another side |
| 1. **Vakrasana**   Sit down stretching your legs forward on the ground. Keep your hands beside your thighs or buttocks. Bend your right leg straight and stretched. Keep one leg raised at 90 degrees. Inhale deeply, later exhale and place the right arm by the outer side of the left knee and hold the left ankle with the right hand. Hold on the position. Repeat another side. |
| **6. Bhujangasana**   - Lie down on your stomach. Raise your trunk and head supported by the palms. Bend your arms at the elbows - Arch your neck and look upward gently. Hold the asana for 5 seconds |
| **7. Dhanurasana**   - Lie down on your stomach with your feet slightly apart, almost parallel to your hips, and place your arms on the side of your body.Slowly, fold your knees up and hold your ankles with your hands. Breathe in and lift your chest off the ground and pull your legs up and stretch it out. Hold for 10 seconds |
| - 1. **Pawana muktasana**   Bend both legs at the knees and rest the thighs against the abdomen, keeping the knees and ankles together. Encircle the knees with both arms, hands clasping opposite elbows. Bend the neck and place the chin on the knees. Continue to maintain the asana, breathing normally. |
| 1. **Kapala bhati**   Close the eyes and relax the whole body. Inhale deeply through both nostrils, expand the chest. Expel the breath with forceful contractions of the abdominal muscles and relax. Do not strain. Continue active/forceful exhalation and passive inhalation.Complete 30 rapid breaths, then take a deep breath and exhale slowly. This is one round of Kapalabhati. Each round shall be followed by deep breathing. Repeat 2 more rounds. |
| 1. **Pranayama and Meditation**   Bramari  Nadishuddi  Kapala bhati |
